# Medical Society of the County of Erie

**Published:** 1895-02

**Authors:** Franklin C. Gram

**Affiliations:** Secretary


### MEDICAL SOCIETY OF THE COUNTY OF ERIE.

Reported by FRANKLIN C. GRAM, M. D., Secretary.

The seventy-fourth annual meeting of the Medical Society of the County of Erie was convened in the rooms of the Buffalo Academy of Medicine, Tuesday, January 8, 1895.

The president, Dr. Wm. H. Gail, of East Aurora, opened the proceedings shortly after 10 o'clock, and called for the minutes of the last semi-annual meeting; also those of the special meeting held August 4, 1894, both of which were read and adopted.

Dr. Geo. W. McPherson, from the committee on membership, reported favorably upon the applications of Drs. N. Victoria Chappell, Albert H. McBeth, G. B. Hepp, Chas. A. Clements Wm. G. Bissell and A. T. O'Hara. They were unanimously elected as members.

Applications for membership were received from Drs. Clara E. Bowen, Evangeline Carroll, John V. Woodruff, Walter N. Kidder, Hiram A. Kendall, Frank H. Field, R. L. Lounsbury, Thomas B. Carpenter, Homer J. Grant and Fred H. Milliner. Referred to the committee on membership.

The secretary read a letter from Dr. S. W. Wetmore and Dr. Mary B. Wetmore, in which they stated that on their removal to California they resigned as members of this society, but having returned they asked to be reinstated. They had previously been members for thirty and fourteen years respectively.

On motion of Dr. Van Peyma they were reinstated without being required to pay an initiation fee.

The president read his annual address.

Reports of committees were then in order. Dr. J. F. Krug submitted the following:

At the last annual meeting of the society, I was elected chairman of the board of censors. I was not present at the meeting and first learned of the fact through the Buffalo Medical Journal. I have not been in my usual health for the past year, was unable to attend to my own business, and should not have accepted the office had I been present at the meeting. For the same reason I have never called a meeting of the board of censors. I did not resign, believing it to be better policy to continue than to leave the office vacant.

I have, however, upon the request of physicians, caused the arrest of several persons for having violated the law regulating the practice of medicine. Two of them were convicted; one was sent to the work-house, as he was not able to pay a fine, and on the other sentence was suspended. The last one whom I had arrested was a Chinese, who could not be found when his case came up for trial. I have been informed that he has left the city. He gave bail for his appearance when wanted for trial. The bail is in the hands of the district attorney. A number of other violations have been reported to me, where recent graduates in medicine have been unmindful of the present law.

A vote of thanks was tendered Dr. Krug for his labors, and an order for $25 was directed to be drawn in his favor to cover the expenses which he had incurred in his prosecutions.

The secretary read the resignation of Dr. A. L. Benedict from the board of censors. The resignation was accepted.

Dr. W. C. CALLANAN, librarian, submitted the following:

I herewith present my annual report for the past year. As you are aware, the library is situated in the building of the University of Buffalo, where it is conveniently located in pleasant surroundings. The librarian of the university, Miss Chappell, carefully attends to this library, and has a record of all the works contained therein. A request for any volume is cheerfully granted. Whilst the library is small as to the number (262), the variety of subjects contained (43) makes it a valuable one for reference.

Its works are divided as follows: On philosophy, 1; statistics, 3; dictionaries and encyclopedias, 5; lectures, 1; periodicals, 110; pathology, 12; practice, 17; insanity, 1; neurology, 11; ophthalmology, 2; respiratory organs, 2; digestive organs, 2; genito-urinary surgery, 3; obstetrics, 7; medical jurisprudence, 4; gynecology, 1; orthopedics, 1; dermatology, 7; pediatrics, 3; infectious diseases, 4; monographs, 4; microscope, 1; bacteriology, 3; urinary analysis, 1; history, 1; anatomy, 1; histology, 4; philosophy, 2; hygiene, 1; public sanitation, 1; materia medica, 5; regional anatomy, 1; general surgery, 11; bone surgery, 2; tumors, 2.

I enumerate the above to show the wide range of subjects that this library covers. With the advance of medical science many new works should be added. The committee appointed at the last meeting to solicit from members donations of money, books or anything of an historical nature pertaining to the history of medicine or of the society, have sent out a circular with the call for this meeting. I hope that the members will respond liberally and thus make this library a mecca for medical knowledge.

In conclusion I would recommend that a book be purchased in which all the names of the deceased members of the society, or as many as can be obtained, should be placed, together with any history or biography. Thus could be formed what might be termed an honor roll, as some of the men who have been members of this society, and who have died, were among the foremost in the profession.

The special committee, appointed for the purpose of entertaining the delegates to the Central New York Medical Association, which met in Buffalo in October, 1894, reported that it did not need the entire appropriation which was granted at the June meeting. The visitors were entertained at luncheon at "The Genesee," at an expense of $42. The new delegates from Erie county who attended that meeting were: Drs. Eli H. Long, F. W. Bartlett, H. G. Bentz, H. T. Carter, A. L. Benedict, Wm. C. Phelps, F. C. Gram, Lillian C. Randall and Jane W. Carroll.

Dr. H. R. Hopkins, from the committee on hygiene, made a verbal report, in which he stated that there had been more activity in hygienic matters during the past year than during the twenty previous. He stated that although no regular meetings had been held, yet its members had worked with a similar committee from the Academy of Medicine, and the results achieved were familiar to all.

The treasurer, Dr. Edward Clark, submitted his annual report, which showed a balance of $287.58 in the treasury. The report was submitted to an auditing committee, composed of Drs. C. A. Ring, S. S. Green and W. W. Potter, who reported that all the items agreed with the vouchers, and recommended that the report be adopted. They also recommended that the usual sum of $50 each be paid to the secretary and treasurer for services during the past year. Both recommendations were adopted.

On motion the secretary was directed to confer with the council of the Buffalo Academy of Medicine, with a view to secure uniform rates of rent for the use of the Academy's rooms whenever required by this society.

The president appointed the following committee to nominate officers for the ensuing year: Drs. Krauss, Hopkins, McPherson, P. W. Van Peyma and Ira C. Brown. The committee reported as follows:

For president, Dr. Frederick W. Bartlett, of Buffalo.

For vice-president, Dr. Justin G. Thompson, of Angola.

For secretary, Dr. Franklin C. Gram, of Buffalo.

For treasurer, Dr. Edward Clark, of Buffalo.

Board of censors—Dr. John B. Coakley, chairman; Drs. Marcel Hartwig, Eli H. Long, Francis T. Metcalfe and Henry Lapp.

Committee on membership—Drs. Geo. W. McPherson, T. M. Johnson and H. E. Hayd.

Delegates to the Medical Society of the State of New York—Drs. J. H. Pryor, Wm. C. Krauss, M. A. Crockett, F. C. Gram, C. C. Frederick and G. W. McPherson.

On motion of Dr. Hopkins, the secretary was directed to cast one ballot for the nominees. This being done the president declared them duly elected.

Dr. Clayton M. Daniels read a paper on Two Peculiar Cases of Hernia. (See page 395.)

Dr. W. H. Woodbury read a paper on Myxedema, its Diagnosis and Treatment, and presented five cases to the society, giving a detailed account of each, and allowing the members to make any personal inquiries or examinations they desired.

Dr. Wm. G. Bissell gave an account of the bacteriological examination in the diagnosis of diphtheria, in a carefully prepared paper, presenting various cultures and mountings, which the members were given an opportunity to examine under the microscope. He gave an especial account of the methods pursued in this particular by the health departments of New York and Buffalo, giving a synopsis of the results in the latter city.

Dr. George Abbott expressed his gratification at the results of this meeting and of the personal benefits he had derived from listening to the scientific part of the program. He stated that, as a remarkable coincidence, he had that morning discovered a piece of diphtheritic membrane coughed up by one of his patients thirty years ago, and which, together with a memoranda, had been concealed in one of his books during that period. He volunteered to give Dr. Bissell a portion of it for experimental purposes, and the offer was accepted with thanks.

Dr. Hopkins stated that seldom had such an array of living subjects been presented by an essayist before this society.

Similar remarks were made by several other members, after which the president extended the thanks of the society to the essayists for their valuable contributions.

Dr. Rochester spoke of the practical benefits he had derived through being enabled to avail himself of the bacteriological examinations made by the Health Department in suspected cases of diphtheria, and also gave a report of a case in which he had used antitoxin with marked benefit.

Dr. Van Peyma invited all out-of-town members to join the Buffalo Academy of Medicine, or, at least, attend its meetings, since the facilities for reaching most of the country towns are now quite good.

Dr. GAIL then introduced the newly-elected president, Dr. Bartlett, to the society. On taking the chair, Dr. Bartlett thanked the members for the distinction shown him, and stated that, as a remarkable coincidence, this was his seventieth birthday, and he was just rounding out his forty-first year of practice. He would endeavor to promote the interests of the society during his incumbency of the office of president and administer its affairs to the best of his ability.

On motion of Dr. KRAUSS, a vote of thanks was tendered the retiring president, and the society adjourned shortly after 1 P. M.

